# Orthorexia Nervosa and Its Associations with Novel Foods and Body Image Concerns

**DOI:** 10.3390/bs15081138

**Published:** 2025-08-21

**Authors:** Mirko Duradoni, Giulia Colombini, Noemi Gori, Andrea Guazzini

**Affiliations:** 1 Department of Human and Social Sciences, Mercatorum University, 50135 Rome, Italy; 2 Department of Education, Literatures, Intercultural Studies, Languages and Psychology, University of Florence, 50121 Florence, Italy; giulia.colombini@unifi.it (G.C.); andrea.guazzini@unifi.it (A.G.); 3 Centre for the Study of Complex Dynamics, University of Florence, 50121 Florence, Italy

**Keywords:** orthorexia nervosa, novel foods, food neophobia, body image, anabolic steroid use

## Abstract

Research on food-related behaviors has increasingly focused on orthorexia nervosa, but the underlying mechanisms are not fully understood, especially with the rise of new types of healthy foods. This study examines the associations between orthorexic tendencies, as measured by the Orthorexia Nervosa Inventory and ORTO-R, and scores on the Food Neophobias Scale, attitudes toward novel foods, body shape concerns, as assessed by the Body Shape Questionnaire, and drive for muscularity, training adherence, and anabolic steroid use, as measured by the Drive for Muscularity Scale. A total of 306 participants (68.3% female; Mage = 35.4 years, SD = 13.7), who were at least 18 old and fluent in Italian, took part in an online, anonymous data collection. The results revealed mixed associations between ON and the perceived attractiveness or intention to consume novel foods. In contrast, no relationship was found between orthorexia and food neophobia. Additionally, orthorexic dimensions were correlated with greater body shape dissatisfaction (r-scores ranging from 0.44 to 0.52, *p* < 0.001) and a drive for muscularity (r-scores ranging from 0.43 to 0.57, *p* < 0.001). Notably, orthorexic scores showed significant positive correlations with thoughts about anabolic steroid use (r-scores ranging from 0.26 to 0.60, *p* < 0.001) and training adherence (r-scores ranging from 0.39 to 0.53, *p* < 0.001) in a subsample of people who regularly exercise. Of examined the predictors, body shape concerns and thoughts about anabolic steroid use (ß ranging from 0.21 to 0.55, and R^2^ ranging from 0.43 to 0.57, *p* < 0.001) were the most robust predictors of orthorexic tendencies. Overall, the findings highlight the complex relationships between orthorexic tendencies, perceptions, and attitudes, as well as body-related concerns, while also providing new insights into their connection to novel foods.

## 1. Introduction

In recent years, research has shown a growing interest in studying nutrition, food, and eating behavior (Almoraie et al., 2025; Grajek et al., 2022; Linardon et al., 2021; Piochi et al., 2025). In this context, the pursuit of health through rigid eating patterns based on perceived healthy and pure diets has received particular attention in the investigation of Orthorexia Nervosa (Cena et al., 2019; Ng et al., 2024).

The term Orthorexia Nervosa (ON) first entered the psychological and psychiatric lexicon at the end of the 20th century, when Bratman (1997) coined the word from the Greek, meaning “proper appetite”. The authors used the expression Orthorexia Nervosa to describe a psychological condition characterized by an obsessive focus on “healthy” eating and practices, as defined by a dietary theory or a varying set of subjective beliefs that certain foods promote health and purity. This core obsessional trait is accompanied by self-imposed inflexible dietary rules, preoccupation with and rigid avoidance of foods considered to be unhealthy, and compulsive behaviors that tend to impair important areas of functioning (e.g., physical health, psychological, social, and academic functioning). As suggested by a recent literature review on the topic (Cena et al., 2019), ON is receiving increasing attention, nevertheless, it still lacks a common definition, accepted diagnostic criteria, and reliable conceptualization. In this regard, some researchers have proposed a bi-dimensional perspective on orthorexic eating behaviors, distinguishing between problematic orthorexia (i.e., ON) and non-pathological healthy orthorexia (HO) (Barrada & Roncero, 2018; Boutin et al., 2024). HO is conceived as a less rigid form of orthorexic eating behaviors, leading to healthy eating behaviors (Barthels et al., 2019). It reflects a genuine interest in healthy eating, characterized by adaptive dietary practices and the integration of these behaviors into one’s identity. Importantly, HO does not exhibit positive associations with psychopathology and may even be negatively correlated with variables such as disordered eating, perfectionism, and obsessive-compulsive traits (Depa et al., 2019). In contrast, ON has been defined as problematic orthorexia, that is a pathological preoccupation with healthy eating, involving rigid dietary rules, punitive reactions to dietary transgressions, and significant impairment in social functioning. It is consistently associated with measures of psychological distress. Notably, HO and ON have been conceptualized as rather distinct, moderately related constructs (Barrada & Roncero, 2018). This distinction is further supported by differences in motivational profiles: while HO is primarily driven by health-related concerns, ON is more strongly associated with motives such as weight control, emotional regulation, and sensory appeal (Depa et al., 2019).

Researchers are currently analyzing the clinical elements that characterize ON and its potential relationships with psychological phenomena related to the eating domain. The issues at present under the lens of investigation include its relationship with food (e.g., food neophobia) and the association between ON and the body, broadly considering the various components of body image.

### 1.1. Orthorexia and Food: Relationships with Traditional and Novel Foods

Eating habits and preferences evolve throughout life (Møller, 2015), and food consumption patterns are subject to change in a delicate balance with environmental conditions (Bracale & Vaccaro, 2020; Eftimov et al., 2020; Lakew et al., 2025). In an ever-changing world, people may choose a variety of diets, with the possibility of interfacing with both traditional and new emerging food products (European Commission, 2023).

In this framework of food-related behavior, food neophobia is defined as an aversion to unfamiliar foods (Babicz-Zielińska, 2006). Some studies have considered it as an orthorexic tendency, particularly related to foods that are considered unhealthy (Bóna et al., 2019; Tragantzopoulou & Giannouli, 2024), as it has sometimes been described as being based on a fear of the artificiality of food, as opposed to the perception of its naturalness and purity (Nicolosi, 2006). Initial theoretical discussions on neophobic traits in orthorexia, like food neophobia in general, attribute them to an evolutionary and automatic response to certain foods (e.g., taste aversion), also linking it to forms of social learning through observing others’ eating behaviors and food preferences (Bóna et al., 2019). On this adaptive basis, some aspects representing non-adaptive dimensions of orthorexia and food neophobic traits may develop, in particular, potential biomedical complications of extremely selective healthy eating patterns and possible concomitant psychological disorders have been examined (Bóna et al., 2019).

Recent studies have linked ON to high harm avoidance and low self-directedness, reflecting anxiety, emotional rigidity, and a strong need for control (Kiss-Leizer & Rigó, 2019). These characteristics may suggest a lower openness to novelty. Specifically, in the context of food, this may translate into lower acceptance of new foods, especially those perceived as unfamiliar or unnatural. However, some research argued that Orthorexia Nervosa can be primarily driven by motives such as affect regulation and control over one’s diet and weight, rather than health food content, distinguishing it from non-pathological healthy orthorexia, which emphasizes the perceived healthiness of food (Depa et al., 2019). Although food neophobia has been proposed as a contributing factor to ON (Koyu et al., 2024; Scarff, 2017), the available empirical evidence is inconsistent, indicating the need for further development of specific theoretical models.

Some studies reported no significant association between food neophobia and ON (Basaran & Ozbek, 2023; Ucar et al., 2018). However, more recent studies suggested a more complex relationship: healthy orthorexia tends to correlate negatively with food neophobia, reflecting greater openness to new foods, while pathological orthorexia shows a positive correlation, likely driven by attempts to control food consumption due to dietary rigidity, especially when uncertain about food content (Koyu et al., 2024).

Nevertheless, certain aspects of ON may suggest a tendency toward potential selective openness toward novel foods, if they align with one’s health values. People with high ON tendencies may be attracted to products promising optimized nutrition, purity, or functionality, such as superfoods, algae-based items, or extracts (Kiss-Leizer & Rigó, 2019). Moreover, the moral or spiritual motivations present in ON could align with the sustainability or ethical framings associated with some novel food technologies (Depa et al., 2019; Kiss-Leizer & Rigó, 2019). Additionally, foods offering precise nutritional profiles or clear traceability may appeal to individuals seeking control and predictability in their diet (Kiss-Leizer & Rigó, 2019).

Based on this mixed theoretical perspective, it might be expected that orthorexia nervosa (ON) may not be linearly associated with food neophobia, particularly regarding novel foods. This is based on the assumption that people with orthorexic tendencies may try new products depicted as healthy options to enhance and fulfill their perceived healthy dietary control.

Thus, preliminary evidence in this area suggests the need for further research, and the current period offers interesting opportunities to continue investigating and clarifying these issues.

In this direction, and taking into account the increasing novelty of food products, research on sustainability and innovation has recently explored novel foods (Monaco et al., 2023; Scaffardi & Formici, 2022). They are foods that have not been consumed to any significant degree by humans in the European Union before 15 May 1997, the year of publication of Regulation 258/97 released by the European Parliament and the Council on novel food products and ingredients (European Commission, 2023). According to the European Commission (2023), novel foods are those that are produced using new production technologies and innovative processes (e.g., UV-treated foods) and may comprise foods traditionally consumed, currently or in the past, outside the European Union, including third countries agricultural products (e.g., chia seeds) or also extracts from existing foods (e.g., Antarctic Krill oil). Other examples are insect products, cell culture-derived foods (e.g., cultured meat, eggs, algae, fungi) (Mazac et al., 2022; Monaco et al., 2023), and nanofoods (i.e., foods produced or processed through nanotechnology or with added nanomaterials (Hebbar et al., 2020; Monaco et al., 2023). Here, a growing body of evidence is currently highlighting the potential benefits of novel foods for sustainability (Dega & Barbhai, 2023; Mazac et al., 2022; Scaffardi & Formici, 2022). Indeed, due to their ingredients, they are described as promising nutritious options for reducing the environmental impact of diets, food production, and food waste, as well as having a positive influence on overall health and well-being (Dega & Barbhai, 2023; Mazac et al., 2022; Sforza, 2022). In this context, the nutritional value and potential health benefits of specific novel foods have attracted increasing scientific attention. For instance, chia seeds are rich in omega-3 fatty acids, fiber, and antioxidants (Sforza, 2022), and have been linked to a reduced risk of cardiovascular disease, type 2 diabetes, and inflammation (Zare et al., 2024). Similarly, baobab pulp is a good source of vitamin C, calcium and dietary fiber, and has reported anti-inflammatory and antimicrobial properties (Abdulwaliyu et al., 2024). Water chestnuts, which have been used in traditional medicine systems, exhibit antioxidant and neuroprotective effects due to their bioactive compound profile (Kumar et al., 2024). Spirulina, a microalga, is notable for its high protein content and nutrient density (Verma et al., 2024). Krill oil, which is rich in bioavailable omega-3s, has shown potential in supporting cardiovascular and cognitive health (Shahidi & Abad, 2024). Finally, edible insects, such as crickets, are emerging as a sustainable source of high-quality protein and essential micronutrients with additional functional properties, including antioxidant and anti-inflammatory activity (Aguilar-Toalá et al., 2022; Indelicato et al., 2025). Together, these novel foods reflect a shift towards healthier and more environmentally sustainable dietary options.

To the best of our knowledge, no studies have yet examined the relationship between orthorexic tendencies and the attractiveness of and intention to eat novel foods, this is therefore among the aims of our work.

### 1.2. Orthorexia and Body: Relationships with Body Image Facets

Extending the field of food-related phenomena to the research of functional and dysfunctional eating patterns, the literature shows mixed results regarding the study of ON and body aspects. On the one hand, according to available diagnostic criteria, orthorexia differs from eating disorders such as anorexia nervosa and bulimia nervosa precisely because it lacks concerns about body weight and shape (Brytek-Matera et al., 2018; Cena et al., 2019). Moreover, it is recognized that people with orthorexic tendencies should not report body image disturbances, a fixation on their physical appearance or show negative attitudes towards their own body that typically characterize eating disorders (e.g., body dissatisfaction, and altered body perceptions) (American Psychiatric Association, 2013; Cena et al., 2019). Indeed, while in classical eating disorders, the drive for an extremely thin body is crucial, in orthorexia the ideal projected onto the body seems to be related only to achieving and maintaining a concept of purity (Brytek-Matera et al., 2018; Ng et al., 2024). In this framework, the conceptualization of orthorexia nervosa described by Bratman (1997) includes some latent features that need to be accounted for. In particular, these comprise a potential form of covert conformism that may encourage people to achieve a socially and culturally accepted model of beauty through their restricted eating behavior, subconsciously following beauty-related social norms (Bratman, 1997). In this vein, Orthorexia has also been described as embedded in an underlying pervasive “social pathology” (Donini et al., 2004; Hanganu-Bresch, 2019, p. 2). This suggests that, in some cases, orthorexia may reflect hidden forms of conformity to society’s dominant aesthetic ideals. In this context, healthy eating may become a socially acceptable way to adhere to these norms and trends without explicitly acknowledging them (Hanganu-Bresch, 2019). Thus, according to this approach, orthorexia may be viewed as deeply rooted in broader social and cultural expectations, functioning as a means of exerting control over one’s life through food, building a personal identity based on dietary purity and moral superiority through healthy habits. However, socially, this could potentially lead to withdrawal from social interactions, while attempting to conform to the illusion of perfect health (Hanganu-Bresch, 2019). Given that the social construction of the ideal beauty in contemporary society frequently associates healthiness with an idealized thin or muscular physical appearance (Bi et al., 2024; Kalender, 2020), recent literature has explored the issue further. Different studies have provided evidence for a potential link between orthorexic tendencies and specific attitudinal and perceptual components of body image, at both explicit and implicit levels. In particular, positive correlates have been found in the following: overweight preoccupation (Pauzé et al., 2021), internalization of thin and athletic ideals (White et al., 2020), investment in physical health (Pauzé et al., 2021), exercise dependence (White et al., 2020), preoccupation towards appearance, implicit muscularity distortion (Pauzé et al., 2021), drive for muscularity (White et al., 2020), and satisfaction with some body parts (Plichta et al., 2019). On the other hand, some studies have more specifically identified overvaluation of weight and shape (Messer et al., 2022), and thinness and muscular internalizations (Tóth-Király et al., 2021) as potential predictors of ON symptomatology.

Considering then that an obsessive focus on healthiness may sometimes overlap with a preoccupation with muscularity, one line of research has explored the association between ON and a focus on muscle appearance, also including possible relationships with Muscle Dysmorphia (MD) (i.e., a fixed preoccupation with muscle appearance that is perceived as not lean or muscular enough (American Psychiatric Association, 2013)). MacPhail and Oberle (2022) summarize two hypothetical associations: (i) a possible direct relationship between ON and MD, as evidenced by positive correlation studies, and (ii) an indirect relationship among them, mediated by some common psychopathological features, such as obsessive-compulsive traits.

In this line, specific research has been carried out also on populations of athletes, fitness, and gym enthusiasts. Here, findings highlight correlations between ON manifestations and physical appearance and frequent exercise (Almeida et al., 2018; Lichtenstein et al., 2017), while orthorexia has also been found to be among the predictors of Muscle Dysmorphia in bodybuilders (Cerea et al., 2018). In addition, some preliminary evidence has observed greater orthorexic tendencies and toward MD among bodybuilders and powerlifters who reported steroid use, at present or in the past (MacPhail & Oberle, 2022).

However, due to the cross-sectional nature of the current studies, the directionality and mechanisms of these associations have yet to be clearly explored. Despite this acknowledgment, the state of the art may generally suggest that the potential role of body image attitudes, including distortions of body shape, and weight and implicit idealizations of a particular body appearance, should be also taken into consideration when assessing obsessive concerns about healthy eating.

Taken together, all these findings highlight the complexity that characterizes orthorexia itself and its interrelationships with various food-related phenomena, both previously studied or new, and recommend the need for further research.

### 1.3. Aims of the Study and Hypotheses Development

The literature reported two main lines of research on Orthorexia Nervosa, namely defining the core characteristics of its relationships with food on the one hand and with the body on the other. Within these research veins, the present study aims to investigate the association between orthorexic tendencies and food-related attitudes and to explore the relationship with body image facets.

#### 1.3.1. Hypotheses on the Association Between ON and Food Attitudes

Firstly, considering the relationship between ON and food, the authors intended to investigate the association between orthorexic tendencies and (i) food neophobia, and (ii) attitudes towards novel foods, to contribute to the understanding of whether orthorexic trends lead to a peculiar focus on well-known foods or the perceived healthiness of novel ones. Given the mixed evidence in the literature outlined above on Food Neophobia and the lack of specific studies on novel foods, it is plausible to hypothesize that people with ON tendencies may exhibit an openness towards novel foods, as they are particularly presented as healthy and nutritious options (Abdulwaliyu et al., 2024; Aguilar-Toalá et al., 2022; Indelicato et al., 2025; Kumar et al., 2024; Shahidi & Abad, 2024; Verma et al., 2024; Zare et al., 2024). This would be aligned and consistent with people’s underlying values and motivations for dietary control, purity, and perceived healthiness (Depa et al., 2019; Kiss-Leizer & Rigó, 2019; Koyu et al., 2024).

The hypotheses are as follows:

**H1.**  *ON is not associated with Food Neophobia;*

**H2.**  *ON is positively associated with the perceived attractiveness of novel foods;*

**H3.**  *ON is positively associated with the intention to consume novel foods.*

While H1 would align with the findings of some authors (Ucar et al., 2018) and contribute to the existing literature on this topic, H2 and H3 would expand scientific knowledge because, to our knowledge, no study has addressed these issues.

#### 1.3.2. Hypotheses on the Association Between ON and Body Image

Secondly, considering the relationship between ON and body, the authors aim to assess the association between orthorexic tendencies and (iii) dimensions of body image concerns in a sample of the general population, and (iv) factors related to the drive for muscularity in a subsample of people regularly engaged in physical activity.

Here, the present study moves along two axes, specifically exploring the relationship with fat-related body image attitudes, and the association with muscular-related attitudes (i.e., focusing on training behaviors and anabolic steroid use), aligning to the latest scientific findings.

The hypotheses are as follows:

**H4.**  *ON will be positively associated with muscular-related attitudes;*

**H5.**  *ON will be positively associated with the use of supplements;*

**H6.**  *ON will be positively associated with training adherence.*

The ultimate aim of this study is to contribute to a deeper and increasingly integrated understanding of orthorexic behavior, both extending previous findings and exploring new avenues.

## 2. Methods

### 2.1. Participants and Procedure

This study included a total sample size of 306 participants, predominantly cisgender females (68.3%) with an average age of 35.4 (SD = 13.7). A non-random, snowball sampling technique was used. We chose this sampling method because, although it is non-probabilistic, it is considered an effective technique to access some populations, promoting participation via one’s own networks of social connections, while also maintaining a varied sample if managed correctly (Ting et al., 2025).

For the data collection Google modules were used, since we could easily create an online questionnaire that we distributed via email, and publicly through various online platforms and social networks, including Instagram, Facebook, WhatsApp, and Telegram. Data collection took place from 7 April to 7 July 2023, both in online and offline contexts (i.e., word of mouth). The participation in the study was completely voluntary, as specified in the dedicated online message and each participant was informed of their right to withdraw from the study at any time. The inclusion criteria were a minimum age of 18 and proficiency in Italian. Given the exploratory nature of the study in the general population, no other inclusion or exclusion criteria were set. All data collected were completely anonymous to ensure confidentiality, in accordance with Italian privacy legislation (Legislative Decree DL-101/2018) and the EU General Data Protection Regulation (2016/679). 

Before recruiting the sample, we defined an adequate sample size based on the type of analysis we hypothesized to perform (i.e., correlations and regression analyses) through a power analysis using G*Power (Version 3.1.9.7) (Faul et al., 2007, 2009). The analysis with the highest requirements of observations was set as the threshold for the sample size to be reached.

For the regression analysis, a sample of 80 participants would ensure a statistical power of 0.80 to detect medium effects (f^2^ = 0.20) (Cohen, 1992, 2013) with a significance level of 0.05. Given that we included 306 participants, the sample sizes can be considered adequate for the purposes of this study. However, of the 306 participants 167 have been considered eligible for a complete modelization. The participants did not receive any incentives or reimbursement for participating in the study.

### 2.2. Measures

To achieve the research objectives, an online questionnaire was created and administered with the help of Google Forms. Basic socio-demographic information (i.e., age and gender) was primarily requested, then the following measures were used, all of which had their validated Italian versions:

Food Neophobia Scale (FNS-R) (Pliner & Hobden, 1992) is a self-report instrument that investigates the individual levels of food neophobia. We used the Italian version of this scale (Guidetti et al., 2018), which consists of 10 items, measured via a 5-point Likert scale, ranging from 1 = strongly disagree to 7 = strongly agree, for which people need their agree with statements related to new food to be tasted. The following item is an example of the scale: “I don’t trust new foods” and “I am afraid of things I have never had before”. The minimum scale score is 10, and the maximum is 70; a higher score underlies higher food neophobia. The psychometric properties and the validity of the Italian version were reported by Guidetti et al. (2018), revealing a very good internal reliability (α = 0.89).

Ad hoc items were created by the authors to investigate perceived attractiveness and intention to eat novel foods, in particular: Chia seeds, Water chestnuts, Spirulina Algae, Baobab pulp, Krill oil, Clean meat, Cricket flour, Edible insects. Participants in the survey were presented with images of these novel foods and asked to rate their agreement with related statements on a 5-point Likert scale (from 1 = “Not at all” to 5 = “Very much”).

For this evaluation, participants were exposed to the same set of images of novel food used in the study of Duradoni et al. (2025). When selecting the set, we aimed to strike a balance between perceived healthiness and novelty. Some novel foods (e.g., spirulina algae, water chestnuts) were chosen for their recognized health benefits and nutritional value, making them particularly relevant for exploring intention and perceived attractiveness in people with an orthorexic focus. Others (e.g., clean meat) were selected primarily for their novelty, aligning with the study’s interest in food neophobia. This selection strategy allowed us to address the core aspects of the research, namely health-oriented food perceptions and responses to new foods, with a coherent stimulus set.

*Orthorexia Nervosa Inventory (ONI)* (Oberle et al., 2021) was employed to assess orthorexic tendencies and behaviors. It has been validated in Italian by Zagaria et al. (2023). It is a three-factor scale with 24 items rated on a 4-point Liker scale, ranging from 1 = not at all to 4 = very. The first dimension (F1), “ONI Behaviors”, assesses behaviors and preoccupation with healthy eating, an example of an item is “Preparing food in the most healthful way is very important in my diet”. The second factor (F1), “ONI Impairments”, examines physical and psychosocial impairments, an example of item is “Whenever I feel sick, family or friends comment that the illness may be because my diet is too restrictive”. The last dimension (F3), “ONI Emotions”, explores emotional distress, an example of an item is “When I stray from my healthy diet, I can only think about what a failure I am”. The minimum scale score is 24, and the maximum is 96; a higher score highlights higher orthorexic tendencies. The psychometric properties and the validity of the Italian version reveal an excellent internal consistency (F1: ω = 0.885; F2: ω = 0.884; F3: ω = 0.913). A potential cutoff score of 72 was proposed by the authors to indicate the presence of, or a high risk for, orthorexic symptoms (Oberle et al., 2021). However, the same authors acknowledged that this threshold is somewhat arbitrary and questionable due to individual variability and the subjective nature of the construct.

ORTO-R (Rogoza & Donini, 2021) is a self-report instrument developed to assess orthorexic tendencies and behaviors. It is a revised and abbreviated version of the ORTO-15 (Donini et al., 2005), created to address issues related to the original scale’s factorial instability and its lack of alignment with the most recent conceptualizations of orthorexia nervosa (ON). The scale consists of 6 items selected from the ORTO-15, identified as the most reliable indicators of ON symptoms. These items are assessed using a 4-point Likert scale (1 = never, 2 = sometimes, 3 = often, 4 = always). The ORTO-R employs a reversed scoring system compared to the ORTO-15, where higher scores indicate a greater tendency toward orthorexic behaviors. An example item from the scale is: “Does thinking about food excessively worry you for more than three hours a day?”. The ORTO-R has been validated in Italian, showing good psychometric properties (ω = 0.75) (Rogoza & Donini, 2021).

Body Shape Questionnaire (BSQ) (Cooper et al., 1987) is a self-report instrument designed to assess body dissatisfaction, specifically related to the feeling of being fat. The BSQ is particularly effective for identifying body dissatisfaction in individuals with or at risk for eating disorders such as bulimia nervosa and anorexia nervosa. It consists of 34 items rated on a 6-point scale (1 = never, 2 = rarely, 3 = sometimes, 4 = often, 5 = very often, 6 = always), where higher scores indicate greater dissatisfaction with body shape. The scale measures body dissatisfaction over a time interval of the past four weeks. An example of an item from the scale is: *“Have you been so worried about your shape that you have been feeling you ought to diet?”*. The total score ranges from 0 to 204, with higher scores reflecting greater body dissatisfaction. A cut-off point of 80 is often used to indicate mild concern with body shape. The psychometric properties of the BSQ-34 have been well-established through numerous studies. In the Italian validation (Marzola et al., 2022) the Cronbach’s alpha coefficient for the BSQ-34 was found to be 0.971 in patients and 0.960 in controls, indicating excellent internal consistency. The test-retest stability in patients was 0.987 (measured with an intraclass correlation coefficient), confirming its reliability over time. After administering the aforementioned instruments to the entire sample, participants were asked a screening question to assess whether they regularly engaged in physical activity. Only those who responded affirmatively were directed to complete the final measurement scale: the Drive for Muscularity Scale (DMS). This subsample consisted of 167 people. To define regular exercise, we adopted the World Health Organization’s recommendations (Bull et al., 2020): engaging in at least 150 to 300 min of moderate-intensity aerobic exercise or 75 to 150 min of high-intensity aerobic exercise per week, or an equivalent combination of both intensity levels spread throughout the week.

Drive for Muscularity Scale (DMS) (McCreary et al., 2004) is a self-report instrument that assesses an individual’s desire for a more muscular body, analyzing attitudes and behaviors related to the drive to increase body mass. The scale is made of 15 items on a 6-point Likert scale ranging from 1 (“Always”) to 6 (“Never”). The scale used in the study is the one developed by Escoto et al. (2013). The questionnaire has a three-factor structure: Attitudes, which assesses attitudes toward muscularity (α = 0.87); Supplement Consumption, which evaluates the use of supplements aimed at increasing muscle mass (α = 0.72); and Training Adherence, which measures commitment to physical training (α = 0.68). This scale was chosen because analyzing supplement use was one of the study’s main objectives, and this specific factor is uniquely captured in this version of the DMS, unlike in other versions. The DMS was only administered to people who regularly exercised because the aim was to investigate how a potential drive for muscularity might influence orthorexic tendencies in male and female exercisers, thereby providing a more comprehensive understanding of the phenomenon.

### 2.3. Statistical Analysis

To explore the associations between orthorexic tendencies and the variables of interest (i.e., food neophobia, perception of novel foods, and body-related concerns), we performed correlation and linear regression analyses among the collected factors, after conducting descriptive analyses and checking for the respect of statistical assumptions of each analysis. We also conducted a mean comparison between sexes using Welch’s t-test or Mann–Whitney U test based on the variables distribution. In the regression analysis, the ONI dimensions (i.e., ONI impairments, behavior, and emotions) and the ORTO-R were set as the dependent variables. R^2^ (i.e., the explained variance) was used as model fit index. The intention to use anabolic steroids was the only variable transformed into a dummy one for analyses purposes. We used SPSS 23 (IBM, 2025) to carry out the analyses.

## 3. Results

First, we calculated the mean and standard deviation for all collected metric variables, presenting these statistics in a sex-fair manner by disaggregating the data for cisgender men and women (the only categories with a sufficient number of observations to ensure robust estimates). For further details, please refer to Table A1 in the Appendix A. Subsequently, we assessed the normality of the distributions to determine the appropriateness of parametric analyses. This was evaluated by examining the skewness and kurtosis values of each variable. Most variables approximated a normal distribution, with skewness and kurtosis values falling within the range of −1 to +1. However, exceptions included the dimensions of the DMS scale (except for DMS attitudes), the “impairments” dimension of the ONI test related to orthorexia, perceived attractiveness, and the intention to consume cricket flour and edible insects. Consequently, we employed non-parametric statistical tests that do not require a normal distribution for these variables that did not meet the normality assumption.

We then assessed the association between the variables using the Pearson coefficient, except for the variables “ONI impairments”, the perceived attractiveness, and the intention to consume cricket flour and edible insects, for which the association was analyzed through Spearman’s Rho due to non-normal distribution.

Table 1 shows mixed results. Significant and positive correlations were found between the perceived attractiveness and intention to eat chia seeds and all the orthorexic measures, while PA and I related to water chestnut showed positive correlations with all measures except for ONI impairments. PA of spirulina algae was positively correlated with ONI behavior, while the intention to eat it was also correlated with ORTO-R scores. Furthermore, the intention to eat baobab pulp positively correlated with behavioral and emotional dimensions of the ONI and the ORTO-R, while its perceived attractiveness was only associated with the ORTO-R. The intention to eat krill oil showed a positive correlation with ONI behavior. Regarding clean meat, its PA correlated with ONI emotions and ORTO-R, whereas the intention to eat it was positively related to all the orthorexic measures. Finally, the PA and I of cricket flour and edible insects showed a positive correlation only with ORTO-R, except for the intention to eat edible insects, which also correlated with ONI behavior. However, the correlations ranged from weak to moderate, reflecting the preliminary nature of the findings, that should be further explored.

We proceeded to explore the degree of association between the variables using the Pearson coefficient, except for the variables “DMS supplement” and “DMS ITEM10: anabolic” for which the association was analyzed through Spearman’s Rho due to a non-normal distribution.

The data presented in Table 2 show no correlations between the different Orthorexia measures considered and Food Neophobia. By contrast, significant and positive correlations were found between Orthorexia and scores on the Body Shape Questionnaire for the whole sample, as well as between Orthorexia and subscales and some specific items of the Drive for Muscularity Scale for a subsample of people reporting regular exercise. This implies that higher values on orthorexic tendencies, specifically higher levels of physical and psychosocial impairments (i.e., ONI impairments), behaviors and preoccupation with healthy eating (i.e., ONI behaviors), and related emotional distress (i.e., ONI emotions), and orthorexic thoughts and behaviors (i.e., ORTO-R) correspond, on average, to higher levels of body dissatisfaction and preoccupation (i.e., BSQ), higher scores in attitudes toward muscularity (i.e., DMS Attitudes), higher use of supplements (i.e., DMS Supplement), higher adherence to training (i.e., DMS Training adherence), and higher levels of thinking about taking anabolic steroids (i.e., DMS item 10). The correlations here ranged from moderate to strong, indicating a substantial association among the collected variables.

To predict orthorexia levels based on the identified predictors through linear regression analysis, we applied a logarithmic transformation to the variables that did not exhibit a normal distribution. The only exception was the variable concerning the intention to use anabolic steroids, which was dichotomized and converted into a dummy variable. This transformation categorized participants into two groups: those who completely ruled out the possibility of using anabolic steroids and those who acknowledged any possibility of their use. As shown in Table 3, higher levels of body dissatisfaction and preoccupation (i.e., BSQ), and thinking about taking anabolic steroids (i.e., DMS item 10) emerge as predictors of orthorexic tendencies in people reporting regular exercise. Given the multiple factors that typically influence human behavior, the effects observed in the present analysis can be considered as meaningful, accounting for between 43% and 57% of the variance in the variables of interest, suggesting a substantial explanatory power.

On the other hand, age, sex, Food Neophobia, attitudes toward muscularity, and training adherence do not show a significant role in the predictive model.

## 4. Discussion

To further advance the understanding of orthorexia nervosa (ON), this study first aimed to investigate the potential association between orthorexic tendencies and receptiveness to novel foods, a factor not addressed in the existing literature.

Moreover, given the lack of a shared consensus on various ON results, the present study aimed to analyze specific dimensions that have produced inconsistent findings in prior research. In particular, we examined the relationship between orthorexic tendencies, food neophobia, and body image, domains that remain theoretically and empirically contentious (Basaran & Ozbek, 2023; Bóna et al., 2019; Cena et al., 2019).

**Figure A1:**
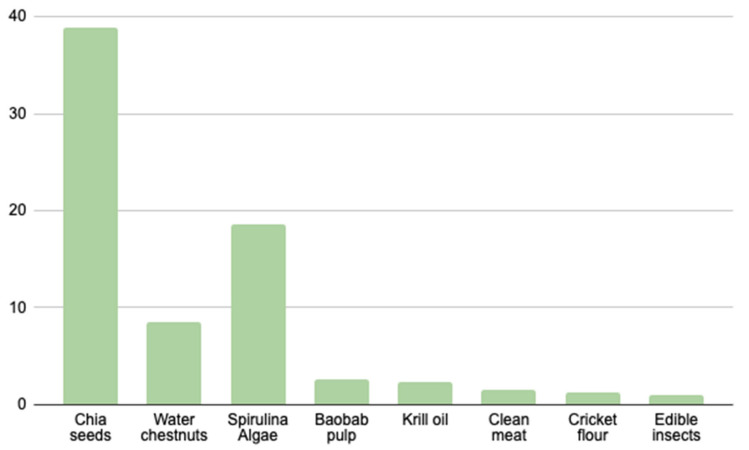
Distribution of actual participants’ consumption of novel foods. The X-axis shows the types of novel foods and the Y-axis shows the mean sum of “yes” responses to actual consumption.

First, the findings showed mixed results regarding the relationship between orthorexic tendencies and the intention to consume novel foods. Generally, significant positive correlations were found between specific dimensions of ONI and ORTO-R measures and perceived attractiveness of and intention to consume chia seeds, water chestnut, and baobab pulp. Similarly, for spirulina algae, krill oil, and clean meat, selective correlations with some ONI dimensions and ORTO-R scores. In contrast, perceptions and intentions regarding cricket flour and edible insects were positively correlated primarily with ORTO-R scores. However, the strength of the correlations ranges from weak to moderate, inviting to be cautious when interpreting the results. Despite this, these preliminary results suggest that orthorexic tendencies may significantly associate with attitudes and intentions toward novel foods, especially when they are perceived as healthy or beneficial to personal well-being. This is particularly relevant given that the existing literature emphasizes that such foods are promoted not only for their potential environmental and functional benefits but also for their nutritional value (Dega & Barbhai, 2023; Mazac et al., 2022). Within this framework, our study provides preliminary evidence that heightened health and food quality concerns may be associated with greater acceptance of new food products. In addition, the data collected on actual novel food consumption (Figure A1 in the Appendix A) closely aligns with the consumption distributions reported in previous studies (e.g., Figure 1 in Duradoni et al., 2025). Specifically, chia seeds, water chestnuts, and spirulina algae emerged as the three most consumed novel foods in both our study and in the research of Duradoni et al. (2025), followed by baobab pulp, krill oil, clean meat, cricket flour, and edible insects. In this specific case, these patterns could stem from perceived healthfulness, given their widely recognized health benefits and awareness of them. Indeed, research has reported on the healthy properties of chia seeds, which are rich in omega-3 fatty acids, fiber, and antioxidants (Zare et al., 2024), as well as those of krill oil (Shahidi & Abad, 2024). Other studies have confirmed the nutritional value of baobab pulp, which is high in fiber and vitamins (Abdulwaliyu et al., 2024), the antioxidant properties of water chestnuts (Kumar et al., 2024), and the high-quality proteins found in spirulina algae (Verma et al., 2024) and edible insects (Aguilar-Toalá et al., 2022; Indelicato et al., 2025). These foods have overall been associated with cardiovascular, metabolic, cognitive, and anti-inflammatory benefits due to their health benefits, nutritional value, and antioxidant and anti-inflammatory bioactive properties (Aguilar-Toalá et al., 2022; Indelicato et al., 2025; Shahidi & Abad, 2024; Zare et al., 2024).

On the other hand, the consumption distribution could also reflect differences in familiarity and availability (e.g., chia seeds are generally more readily available than clean meat).

Second, no significant correlations were observed between orthorexic tendencies and food neophobia. These results support our initial hypothesis and are consistent with the existing body of literature that has not identified a significant correlation between food neophobia and orthorexia nervosa (Basaran & Ozbek, 2023; Ucar et al., 2018). These findings may suggest that people with orthorexic tendencies may prioritize perceived healthiness over familiarity when making food choices. However, only a few studies have investigated the specific association between orthorexia and healthy foods to date. Osowiecka and Myszkowska-Ryciak (2020), for example, found that people with higher tendencies of orthorexia nervosa reported healthier food choices (e.g., increased consumption of whole wheat cereals, fruits, and vegetables), greater engagement in health-focused behaviors (e.g., shopping in health food stores, practicing dietary restrictions and reduced alcohol intake) and a stronger inclination to promote their dietary practices to others. In another study, Varga et al. (2014) observed weak associations between orthorexia risk and the consumption of certain novel foods, such as goji berries, ginseng, and amaranth. These initial results highlight the need for further research into the psychological and behavioral correlates of the association between ON and the consumption of new healthy foods.

Regarding body-related aspects, the results revealed positive associations between orthorexic tendencies and Body Shape Questionnaire (BSQ) scores across the entire sample. Specifically, body dissatisfaction emerged as a significant predictor of orthorexic tendencies, whereas age, gender and food neophobia did not significantly predict orthorexic behaviors.

When examining the subscales of the Drive for Muscularity Scale (DMS), note that the analyses were conducted with participants who regularly engage in physical activity. Within this subgroup, contemplation of anabolic steroid use was found to be a significant predictor of orthorexic tendencies. However, attitudes toward muscularity and adherence to training were not found to be significant predictors of orthorexic behaviors. The results highlighted moderate to strong correlations and a considerable explanation power of the examined predictors.

The findings of this study are consistent with existing literature that identifies a relationship between orthorexia nervosa (ON) and body-related factors, contributing to the ongoing scientific discussion in this field. In particular, the positive associations observed between orthorexic tendencies, body dissatisfaction (BSQ) and certain aspects of the drive for muscularity (DMS), including supplement use and contemplation of anabolic steroid use, appear to support our initial hypothesis. These results align with previous studies that have highlighted a link between ON, internalization of aesthetic ideals (e.g., β = 0.27, *p* = 0.01, partial r = 0.26, for both thin and low body fat internalization), and behavioral drive for muscularity (β = 0.37, *p* = 0.002, partial r = 0.31), even after controlling for eating disorder symptoms and exercise addiction (F(2, 88) = 14.37, *p* < 0.001, R^2^ = 0.51) (White et al., 2020), and average muscularity distortion (β = 5.360, *p* = 0.021) (Pauzé et al., 2021).

These findings suggest that body image may play a significant role in the development or maintenance of orthorexic tendencies. However, the DMS-related results may have been influenced by the characteristics of the analyzed subsample, which consisted of people who regularly engage in physical activity (e.g., gym-goers). This raises the possibility that there may be multiple subpopulations of people with orthorexic tendencies, each with a different body-related profile. For instance, people who are highly focused on food purity and health would not typically be expected to consider steroid use; however, a subgroup with both orthorexic and performance-oriented characteristics may adopt a different mindset, placing greater emphasis on muscularity and physical performance. This hypothesis underscores the need for further research to explore potential heterogeneity within orthorexic presentations. 

Furthermore, the results also underscored a significant association between ONI emotions, body shape concerns, and anabolic steroid use. In this sense, the emotional distress component of ON could be further explored to better unravel the psychosocial aspects of this condition. For example, recent findings by Raffone et al. (2025) highlighted the role guilt in influencing eating disorders differently. Our study supports the importance of emotional distress in food-related behavior, while also suggesting the need for more targeted research on the specific mediators of this distress. Guilt, in particular, could help understanding the importance of emotional distress as one of the potential linking factors of maladaptive control strategies, body-related perfectionism, and judgment dynamics across various eating disorder patterns.

Overall, the present findings may contribute to the understanding of orthorexia by suggesting that perceptions of new foods and body-related concerns could be relevant factors. Although these aspects are not currently included in the standard conceptualization, their significant potential role could be explored further, especially given the ongoing debate and controversies surrounding the definition, conceptualization, and operationalization of the ON construct (e.g., Cena et al., 2019; Pontillo et al., 2022; Strahler & Stark, 2020).

In general, these findings underscore the need for more research in this area to better understand the motivations that may drive orthorexic behaviors, both potentially considering attitudes towards novel phenomena in the food area as well as in its link to one’s relationship to body. This could contribute to clarifying the current scientific debate on the topic and help refine future studies and clinical approaches. In particular, the results underscore the importance of recognizing subpopulations with unique psychological and behavioral profiles and needs. This could support the development of more targeted prevention efforts and personalized intervention strategies, ultimately enhancing the efficacy of health promotion in populations at risk for dysfunctional eating patterns.

### 4.1. Limitations of the Study

While this study provides useful insights into orthorexic tendencies and their associations with other psychological and behavioral variables related to food and body domains, some limitations should be acknowledged. First, the composition of the sample may have influenced the generalizability of the findings. The slight predominance of female participants does not rule out a potential gender bias, even if the analysis of gender differences in orthorexic tendencies were beyond the scope of this paper. Second, the non-random sampling does not exclude a self-selection bias, thereby limiting the representativity and generalizability of the results. In this regard, it is worth noting that the recruitment procedure based on snowball sampling and online data collection is common in this field of research. However, it may contribute to selection bias and limit sample diversity. Additionally, analyzing a subsample of people who regularly exercise may have introduced some degree of selection bias and analytical heterogeneity.

Third, given the exploratory nature of this study, we only set a minimum age of 18 and proficiency in Italian as inclusion criteria, thereby potentially increasing the heterogeneity of the sample. Future studies could consider applying more specific inclusion and exclusion criteria to address this limitation and explore orthorexic behavior and facets more specifically in subpopulations (e.g., people who perform specific sports or professions, such as dancers; people with specific dietary patterns, such as vegetarians, and food restraints like gluten intolerance or inflammatory bowel syndrome).

Moreover, the cross-sectional design of the study limits the possibility of establishing causal relationships between the variables explored. Since all data were collected at a single time point, the directionality of the associations remains unclear. Longitudinal research would allow for a better understanding of how these relationships can evolve over time and whether certain psychological traits or behaviors may predict the development of orthorexic patterns. In this regard, we did not provide baseline characteristics to complement our cross-sectional data, in terms of socio-demographic information (e.g., ethnicity, work or study status) or health-related information, such as BMI. Future research should include such baseline data, as it could be important for detecting potential changes over time and for better describing the sample and its subgroups.

Furthermore, we did not apply corrections to control for the increased familywise error rate across the multiple statistical analyses conducted. We consider the present findings as preliminary and encourage future studies to further exploration of these results in future studies.

Another methodological limitation concerns the exclusive reliance on self-report measures. Although these tools are commonly used and offer practical advantages, they are susceptible to a range of response biases that may affect the accuracy and reliability of the results, for example due to people’s tendency to provide socially acceptable answers, different introspective abilities or recall bias in providing self-assessed reports. Additionally, the internal reliability of the Supplement Consumption factor is acceptable, while the Training Adherence factor is at the threshold of acceptability. Therefore, the related results should be interpreted with caution. Moreover, another limitation could concern the dichotomization of the anabolic steroid use factor, as it may reduce the complexity of the construct, which may be continuous.

The results do not account for the influence of potential intervening variables. For instance, the study did not analyze perfectionistic tendencies, which could affect the relationship between body-related concerns and orthorexic tendencies. Previous research has indeed reported perfectionism (Dönmez, 2024) as well as metacognitive abilities as relevant in predicting orthorexia (Kılıçaslan, 2025). Additionally, the study did not explore the subjective motivations of participants for engaging in orthorexic behaviors. Understanding these underlying psychological drivers may be important as they could influence or mediate the observed relationships with variables such as food neophobia or body-related attitudes. Furthermore, future studies could examine the influence of cultural factors, which were not present in this analysis. Indeed, some recent research has shown that specific social contexts (Dönmez, 2024) and cultures can impact orthorexic tendencies (Cobzeanu et al., 2025; Brytek-Matera et al., 2020, 2022).

Moreover, the absence of control for potential confounding variables, such as exercise frequency and body mass index (BMI), represents an additional aspect that may have influenced the observed relationships and should be considered when interpreting the findings.

Despite the current limitations, it should be acknowledged that we incorporated two widely recognized measures from the literature to strengthen the present research design on the construct of orthorexia nervosa, namely the ONI and ORTO-R measures. The two tools differ both in their dimensional structure, one being monodimensional (i.e., ORTO-R) and one the other multidimensional (i.e., ONI), and in terms of publication recency. This strategy enabled us to address the diversity in how ON is operationalized across studies. As in previous research (Duradoni et al., 2023), employing both measures allowed us to examine whether the relationships with ON-related variables may vary also depending on the measurement tool used.

### 4.2. Potential Impact and Future Perspectives

The present study contributes to the current understanding of orthorexia nervosa by providing new empirical data on its relationship with food-related attitudes, particularly toward novel foods, an area that remains underexplored in current literature. From a theoretical standpoint, the findings extend previous knowledge by highlighting a potential link between orthorexic tendencies, attitudes toward novel foods, and body-related issues. Recognizing the associations that emerged may open new avenues for broadening the current theoretical understanding of orthorexia, expanding it beyond rigid dietary patterns solely, and also taking into account its distinct manifestations across different population subgroups.

From an applied perspective, these insights could be useful for designing targeted interventions for people with orthorexic tendencies, particularly in clinical settings where early detection and personalized approaches are essential.

Furthermore, the study provides preliminary support for the idea that distinct subpopulations may exist within the orthorexic spectrum, each characterized by specific profiles. This finding is consistent with the broader understanding of eating disorders, in which heterogeneity in symptom expression is often observed despite the presence of core shared features (Williamson et al., 2004). Once more, pinpointing particular subtypes might be crucial for facilitating more customized interventions.

Since this study cannot draw inferences about causal relationships, another important direction for future research concerns the nosological status of orthorexia nervosa. Further investigation is needed to determine whether orthorexia constitutes a distinct clinical entity as initially described (Bratman, 1997); a prodromal stage of other eating disorders; or possibly even a residual outcome of previously diagnosed conditions, in line with the questions posed by recent studies (Łucka et al., 2019). Longitudinal and developmental studies could help clarify these trajectories and refine classification efforts.

Furthermore, future studies could further explore the potential increasing impact of social media on health-related behaviors (Kuş et al., 2025; Usta Ulutaş & Okan Bakır, 2025) regarding ON. Although this information was not analyzed in the present study, it could be valuable for further understanding the underlying mechanisms of the association between ON and body-related concerns, as well as the social influence dynamics on dietary habits regarding healthy food.

Finally, in line with an ethno-psychiatric perspective (Gadit, 2003) and the results of studies in the field (Brytek-Matera et al., 2022; Gramaglia et al., 2019), future research should explore and refine the definition of orthorexia nervosa by considering the differential impact of cultural and social influences. As the risk of orthorexia nervosa (ON) is increasing (Gortat et al., 2021), it is necessary to further investigate its cross-cultural dimensions in order to inform the development of culturally sensitive assessment tools and psychological interventions.

## 5. Conclusions

The present study sheds new light on the psychological and behavioral correlates of orthorexic tendencies. Specifically, the results revealed significant positive correlations between orthorexic tendencies and the perceived attractiveness of, and intention to consume various novel foods, particularly those generally considered healthy. Additionally, no significant associations were found between orthorexia and food neophobia, suggesting that the rejection of unfamiliar foods may not be a defining characteristic of orthorexic behavior. Furthermore, orthorexic tendencies were found to be associated with body-related variables, including body dissatisfaction, and the drive for muscularity, adherence to training regimens, and thoughts about using anabolic steroids, among a group of people who regularly exercise. These findings suggest the potential importance of appearance-oriented motivations and highlight a possible link between orthorexia and broader body image concerns.

Overall, this study underscores the need for further research on these dimensions, particularly on potential subtypes within the orthorexic spectrum, to improve understanding of the phenomenon, contribute to the current scientific debate, and help develop targeted recognition and intervention strategies.

## Figures and Tables

**Table 1:** Correlations between novel foods and orthorexia measures.

Variables	ONI Impairments ^✤^	ONI Behavior	ONI Emotions	ORTO-R
Chia seeds: PA	0.15 **	0.33 ***	0.23 ***	0.22 ***
Chia seeds: I	0.19 ***	0.31 ***	0.24 ***	0.22 ***
Water chestnut: PA	−0.06	0.13 *	0.14 *	0.14 *
Water chestnut: I	−0.03	0.11 *	0.12 *	0.13 *
Spirulina algae: PA	−0.08	0.13 *	0.07	0.10
Spirulina algae: I	−0.08	0.13 *	0.08	0.12 *
Baobab pulp: PA	−0.02	0.10	0.08	0.11 *
Baobab pulp: I	0.03	0.21 ***	0.12 *	0.19 ***
Krill oil: PA	−0.05	0.06	−0.04	0.03
Krill oil: I	−0.01	0.12 *	0.02	0.08
Clean meat: PA	0.09	0.07	0.18 **	0.21 ***
Clean meat: I	0.16 **	0.17 **	0.22 ***	0.27 ***
Cricket flour: PA ^✤^	−0.01	0.01	0.08	0.14 *
Cricket flour: I ^✤^	0.04	0.05	0.09	0.17 **
Edible insects: PA ^✤^	0.01	0.11	0.05	0.12 *
Edible insects: I ^✤^	0.04	0.14 *	0.06	0.14 *

Note: N = 306; PA = Perceived Attractiveness; I = Intention to consume; ONI = Orthorexia Nervosa Inventory; ^✤^ = the association with this variable was analyzed through Spearman’s Rho due to a non-normal distribution; *** = *p* < 0.001; ** = *p* < 0.01; * = *p* < 0.05.

**Table 2:** Correlations matrix among the collected variables.

Variables	FNSR	BSQ	DMS Attitudes °	DMS Supplement °^✤^	DMS ITEM10: Anabolic °^✤^	DMS Training Adherence °
ONI impairments ^✤^	0.07	0.45 ***	0.43 ***	0.40 ***	0.44 ***	0.39 ***
ONI behavior	−0.04	0.26 ***	0.54 ***	0.46 ***	0.52 ***	0.49 ***
ONI emotions	−0.06	0.53 ***	0.57 ***	0.43 ***	0.47 ***	0.53 ***
ORTO-R	−0.07	0.60 ***	0.50 ***	0.37 ***	0.45 ***	0.45 ***

Note: N = 306 (total sample); **°**: N = 167 (people regularly exercising); ONI = Orthorexia Nervosa Inventory; FNSR = Food Neophobia Scale; BSQ = Body Shape Questionnaire; DMS = Drive for Muscularity; ^✤^: the association with this variable was analyzed through Spearman’s Rho due to a non-normal distribution; *** = *p* < 0.001.

**Table 3:** Regression analyses among the collected variables and orthorexia measures.

Variables	Age	Sex	FNSR	BSQ	DMS Attitudes °	DMS ITEM10: Anabolic °^॥^	DMS Training Adherence °	R^2^	F (7;160)
ONI impairments ^✤^	0.07	−0.01	0.07	0.35 ***	0.13	0.32 ***	0.12	0.43	18.55 (*p* < 0.001)
ONI behavior	0.20 **	−0.02	−0.06	0.21 **	0.04	0.41 ***	0.29 ***	0.48	22.43 (*p* < 0.001)
ONI emotions	0.04	−0.07	−0.04	0.45 ***	0.14	0.27 ***	0.12	0.53	26.88 (*p* < 0.001)
ORTO-R	0.01	−0.05	−0.07	0.55 ***	0.08	0.25 ***	0.10	0.57	28.56 (*p* < 0.001)

Note: ° N = 161 (cisgender females and males only); *** = *p* < 0.001; ** = *p* < 0.01; **^॥^** = dummy variable; ^✤^ = log-transformed variable; ONI = Orthorexia Nervosa Inventory; FNSR = Food Neophobia Scale; BSQ = Body Shape Questionnaire; DMS = Drive for Muscularity.

## Data Availability

The data presented in this study are available on request from the corresponding author.

## Appendix A

**Table A1:** Descriptive statistics of the collected variables disaggregated by sex.

Variable	Total Sample N = 306	Cis. Women N = 209	Cis. Men N = 86	Comparison by Sex	
	M (s.d)	M (s.d)	M (s.d)	t/U	d
ONI impairments ^✤^	14.00 [8.00]	14.00 [8.00]	15.00 [9.00]	9676	0.08
ONI behavior	17.98 (6.77)	17.24 (6.38)	19.57 (7.46)	2.53 *	0.34
ONI emotions	9.26 (4.57)	8.87 (4.46)	10.17 (4.78)	2.18 *	0.28
ORTO-R	15.22 (4.71)	14.93 (4.61)	15.58 (4.81)	1.07	0.14
FNSR	16.17 (5.02)	16.19 (8.12)	15.87 (4.73)	−0.51	−0.06
BSQ	41.10 (18.34)	43.59 (18.53)	35.84 (16.86)	−3.49 ***	−0.44
DMS attitudes °	18.02 (9.17)	16.24 (8.12)	22.35 (10.06)	3.82 ***	0.67
DMS supplement °^✤^	7.00 [5.00]	6.00 [4.00]	9.50 [9.00]	4329 ***	0.52
DMS ITEM10: anabolic °^✤^	1.00 [0.00]	1.00 [0.00]	1.00 [2.00]	3680 ***	0.30
DMS training adherence °	11.31 (6.10)	10.09 (5.62)	13.81 (6.30)	3.62 ***	0.62
Chia seeds: PA	2.74 (1.16)	2.87 (1.14)	2.52 (1.19)	−2.28 *	−0.29
Chia seeds: I	2.97 (1.23)	3.11 (1.18)	2.81 (1.31)	−1.79	−0.23
Water chestnut: PA	2.32 (1.05)	2.35 (1.09)	2.33 (0.99)	−0.18	−0.02
Water chestnut: I	2.44 (1.10)	2.49 (1.13)	2.44 (1.02)	−0.34	−0.04
Spirulina algae: PA	2.55 (1.29)	2.66 (1.29)	2.38 (1.24)	−1.72	−0.22
Spirulina algae: I	2.60 (1.28)	2.71 (1.27)	2.43 (1.27)	−1.71	−0.22
Baobab pulp: PA	1.89 (0.94)	1.89 (0.94)	1.97 (0.95)	0.62	0.08
Baobab pulp: I	2.01 (1.04)	2.00 (1.02)	2.12 (1.11)	0.87	0.11
Krill oil: PA	1.77 (0.93)	1.73 (0.87)	1.97 (1.07)	1.83	0.24
Krill oil: I	1.88 (1.00)	1.82 (0.93)	2.10 (1.14)	2.07 *	0.28
Clean meat: PA	1.88 (1.10)	1.86 (1.09)	1.99 (1.15)	0.91	0.12
Clean meat: I	1.98 (1.15)	1.92 (1.12)	2.20 (1.24)	1.80	0.24
Cricket flour: PA ^✤^	1.00 [1.00]	1.00 [1.00]	1.00 [2.00]	10,644 **	0.18
Cricket flour: I ^✤^	1.00 [1.00]	1.00 [1.00]	1.00 [2.00]	10,740 ***	0.20
Edible insects: PA ^✤^	1.00 [0.00]	1.00 [0.00]	1.00 [2.00]	10,498 ***	0.17
Edible insects: I ^✤^	1.00 [1.00]	1.00 [0.00]	1.00 [1.00]	10,900 ***	0.21

Note: °: N = 167 for the total sample (52 cisgender men, 109 cisgender women); ONI = Orthorexia Nervosa Inventory; DMS = Drive for Muscularity; FNSR = Food Neophobia Scale; BSQ = Body Shape Questionnaire; PA = Perceived Attractiveness; I = Intention to consume; t = Welch’s t-test; U = Mann-Whitney U test; ^✤^ = variable described with median and interquartile range and analyzed with non-parametric test due to a non-normal distribution; […] = interquartile range. *** = *p* < 0.001; ** = *p* < 0.01; * = *p* < 0.05.
